# Inbreeding and outbreeding depression in *Stylidium hispidum*: implications for mixing seed sources for ecological restoration

**DOI:** 10.1002/ece3.302

**Published:** 2012-08-07

**Authors:** Kristina M Hufford, Siegfried L Krauss, Erik J Veneklaas

**Affiliations:** 1 Kings Park and Botanic Garden, Botanic Gardens and Parks AuthorityWestern Australia, Australia; 2 School of Plant Biology, The University of Western AustraliaWestern Australia, Australia; 3 Department of Ecosystem Science and Management, University of WyomingLaramie, Wyoming

**Keywords:** AFLP, conservation, hybrid fitness, optimal outcrossing, restoration genetics

## Abstract

The benefits of composite rather than local seed provenances for ecological restoration have recently been argued, largely on the basis of maximizing evolutionary potential. However, these arguments have downplayed the potentially negative consequences of outbreeding depression once mixed provenances interbreed. In this study, we compared intraspecific F1 hybrid performance and molecular marker differentiation among four populations of *Stylidium hispidum*, a species endemic to Southwestern Australia. Multivariate ordination of 134 AFLP markers analyzed genetic structure and detected two clusters of paired sites that diverged significantly for marker variation along a latitudinal boundary. To test for outbreeding depression and to determine the consequences of molecular population divergence for hybrid fitness, we conducted controlled pollinations and studied germination and survival for three cross categories (within-population crosses, short- and long-distance F1 hybrids) for paired sites distributed within and between the two genetically differentiated regions. We found evidence of outbreeding depression in long-distance hybrids (111–124 km), and inbreeding depression among progeny of within-population crosses, relative to short-distance (3–10 km) hybrids, suggesting an intermediate optimal outcrossing distance in this species. These results are discussed in light of the evolutionary consequences of mixing seed sources for biodiversity restoration.

## Introduction

The spatial distribution of genetic diversity and the genetic distance between interbreeding individuals can have significant consequences for the ecological restoration of native plant communities (Keller et al. 2000; Bischoff et al. 2010). Restoration commonly requires the introduction of large quantities of seeds, and available seed stock may be derived from limited sources or long distances (Mortlock 2000; Jones 2003). Genetically depauperate plant material increases the potential for inbred seed stock, whereas divergent source populations may reflect adaptation to environments far removed from restoration sites (Edmands 2007; Broadhurst et al. 2008). If population fitness and long-term sustainability are among the goals of ecological restoration, the risks of inbreeding and outbreeding depression must be considered when selecting seed sources for restoration sites (Rice and Knapp 2000; McKay et al. 2005).

Among populations of native plant species, there is considerable evidence for an optimal outcrossing distance for progeny fitness that minimizes both inbreeding and outbreeding depression (Waser 1993; Schierup and Christiansen 1996; Montalvo and Ellstrand 2001). Mating at short distances often results in inbreeding due to increased relatedness of neighboring individuals. The negative consequences of inbreeding for plant fitness, such as a 50% reduction in heterozygosity each generation with complete selfing, are well documented (Charlesworth and Charlesworth 1987; Byers and Waller 1999; Carr and Dudash 2003). In contrast, mating between genetically divergent individuals can result in a decline in progeny fitness resulting from outbreeding depression (Templeton 1986). Outbreeding depression is caused by one of two possible mechanisms. The first is an “extrinsic” or environmental mechanism where genes fixed for locally adapted alleles are diluted by half in hybrid progeny (Hufford and Mazer 2003). The second is an “intrinsic” (genetic) mechanism resulting from intraspecific chromosomal variation or the loss of coadapted gene combinations (Edmands 2007; Frankham et al. 2011). In the second case, outbreeding depression may not be apparent until the second (F2) generation of hybridization or later, when recombination first occurs (Edmands 2007).

To reduce the risk of inbreeding and outbreeding depression in restored populations, current guidelines for ecological restoration generally recommend introductions of diverse, “local” genotypes to preserve biodiversity and retain adaptation to environmental conditions (McKay et al. 2005; Bischoff et al. 2006; Krauss and He 2006). The scale and extent of local adaptation, however, are often unknown. In these circumstances, molecular markers may serve as tools to detect the scale of divergence and predict boundaries across which population fitness will decline if seed sources are combined for restoration (Knapp and Rice 1996; Bussell et al. 2006). In 2004, Krauss and Koch proposed a method whereby spatial autocorrelation analysis of the association of marker diversity and geographic coordinates was used to determine the distance among samples where genetic correlation is no longer significant. This distance forms a “patch size” diameter within which seeds representing relatively low levels of divergence may be transferred (Krauss et al. 2005). Using a different method, Bussell et al. (2006) compared genetic marker differentiation among populations for multiple species, and grouped species into three categories of narrow, local, and regional provenance based on an overall mean of population differentiation. Non-overlapping clusters of populations within each species were used to predict boundaries for seed transfer. While these methods often remain untested, there is evidence that genetic measures of population divergence can predict reproductive compatibility and subsequent progeny fitness (Edmands 2002). Questions arise, however, when molecular markers are substituted for studies of adaptive differentiation. Most molecular marker data reflect random genetic processes, such as genetic drift and may not correlate with heritable genetic variation (Reed and Frankham 2001; Latta 2004).

Recently, investigators have begun to question the use of “local” provenance in restoration practice. Broadhurst et al. (2008) argue that the reliance on local provenance greatly increases the likelihood of low quality, inbred propagules in highly fragmented landscapes. In these cases, seeds sourced from large populations at greater distances may improve restoration outcomes and minimize the risk of overharvesting remnant, neighboring sites. There are also concerns that existing guidelines focus on germplasm preservation rather than maintaining the evolutionary potential of populations (Broadhurst et al. 2008; Sgrò et al. 2011). Genetic variation is required for species persistence in heterogeneous environments that vary in space and time (Rice and Emery 2003), and local genotypes may no longer be the best source material in light of anthropogenic disturbance and climate change (Sgrò et al. 2011). As an alternative to local provenance zones, the use of a “composite” mix has been recommended, with the objective to combine germplasm from ecologically similar sites in proportion to rates of dispersal for each distance class, including long-distance sources well beyond local provenance ranges (Broadhurst et al. 2008). Consequently, seed mixes would represent combinations of potentially highly divergent parent populations, raising the risk of outbreeding depression in hybrid progeny. A recent review of the available data for outbreeding depression and the probability for its occurrence, however, suggested that concerns about outbreeding in natural populations may be exaggerated (Frankham et al. 2011).

While the avoidance of inbreeding in reintroduction programs is a significant concern, and multiple publications argue in favor of genetic diversity of introduced sources, few studies have tested genetic structure and the consequences of seed mixing in restoration relative to the potential impacts of outbreeding depression (Forrest et al. 2011; Goto et al. 2011). To assess the risks of outbreeding depression and the significance of local provenance, experimental evaluation of intraspecific hybrid performance is needed, as well as trials to compare hybrid performance with estimates of population divergence (Edmands 2002). In this study, we examined molecular marker differentiation and F1 hybrid performance among populations of *Stylidium hispidum*, a species endemic to Southwestern Australia. We analyzed genetic diversity and population structure for four sites distributed between the northern and southern extent of the species range. At the same time, we conducted intraspecific crosses and compared F1 hybrids with parental genotypes for measures of early (germination and survival) fitness in a controlled environment. As a result, we were able to (1) test for significant population genetic divergence among sites, (2) contrast the performance of hybrid progeny relative to parental population fitness and compare performance with estimates of genetic divergence, and (3) predict the potential for outbreeding depression to occur in restored populations of this species established from mixed provenance seed.

## Materials and Methods

### Study species and sites

Southwest Western Australia is a global biodiversity hotspot, in which high levels of endemism (68% of the ≥3600 vascular plant species) occur in a landscape where greater than 70% of the natural vegetation has been altered or lost due to land development (Hopper and Gioia 2004). In addition to agricultural and urban development, the region is also a center for natural resource extraction, and native floras are heavily impacted by a number of mining operations. In the northern Jarrah (*Eucalyptus marginata*) forest alone, more than 550 hectares of post-mined land are restored each year (Bell and Hobbs 2007).

*Stylidium hispidum* Lindl. (Stylidiaceae), or white butterfly triggerplant, is a perennial species endemic to the northern *E. marginata* forests of Southwest Western Australia. Plants have a basal rosette growth form and produce one or more flowering racemes each spring. The style and filaments of *S. hispidum* flowers are fused to form a column that is “triggered” when an insect, such as a native bee lands on the petals (Erickson 1958; Armbruster and Muchhala 2009). Flowers are protandrous, and the trigger either deposits pollen if flowers are in male phase or adheres cross pollen carried by the visiting insect if flowers are in female phase. Seeds have no appendage to facilitate wind dispersal and are primarily gravity dispersed. Perennial species in the Stylidiaceae are often self-incompatible, although there is no evidence for pre-zygotic incompatibility (Burbidge and James 1991). Instead, recessive lethal genes and cytogenetic factors, including some evidence for chromosome variation within species, are proposed as a mechanism for post-zygotic incompatibility (James 1979; Coates et al. 2003). Early karyotype research determined that *S. hispidum* has a diploid chromosome number (*n* = 14; James 1979).

**Figure 1:**
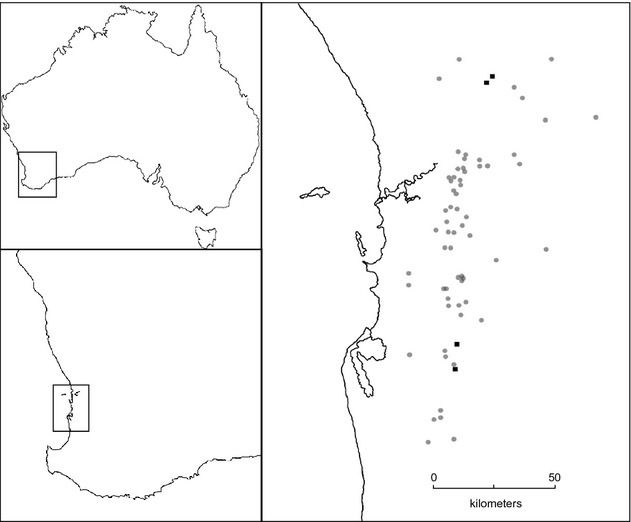
Distribution of *Stylidium hispidum* in Western Australia. Collection sites are identified by black squares and additional known species locations are indicated by gray circles (Western Australian Herbarium 1998).

The distribution of *S. hispidum* is primarily along the Darling Fault, which forms the western edge of the Yilgarn Craton (Fig. 1). We collected leaf samples for genetic analyses from a minimum of 33 individuals from each of two northern sites in Avon National Park and two southern sites in Dwellingup State Forest (Fig. 1, Table 1). Populations of *S. hispidum* at these sites occur in a patchy distribution on highly weathered, lateritic soils in *E. marginata* forest. Targeted populations represented a minimum of several hundred individuals. Regional climate is dry Mediterranean type with hot summers and cool, wet winters and the collection sites span a cline of approximately 663 mm mean annual rainfall in the north to 1240 mm mean annual rainfall in the south (Bureau of Meteorology, Perth, Western Australia). The two southern sites are located near two bauxite mines operated by Alcoa World Alumina Australia (Koch and Samsa 2007) and represent potential source populations for nearby mine site rehabilitation.

**Table 1:** Population information, geographic coordinates (decimal degrees), and genetic diversity indices for AFLP data analyzed for the four experimental populations of *Stylidium hispidum*. Values include population sample size (N), the proportion of polymorphic loci (PLP), and population-level gene diversity (*Hj*)

Region	Population	ID	Latitude	Longitude	*N*	PLP	*Hj*
North	Avon Valley National Park 1	AV1	−31.5621	116.1818	33	89.6	0.27780
	Avon Valley National Park 2	AV2	−31.5849	116.1595	37	83.6	0.27764
South	Dwellingup State Forest (Torrens Road)	TOR	−32.5860	116.0472	33	76.1	0.23848
	Dwellingup State Forest (Del Park Road)	CPC	−32.6772	116.0403	32	76.9	0.25126

### DNA extraction and molecular marker analyses

Fresh leaf or bud samples were preserved on ice in the field and stored at −80°C prior to DNA extraction. Total genomic DNA was extracted using the methods of Wagner et al. (1987) with modification (Byrne et al. 2001). Namely, whole buds or small quantities (5–7) of individual leaves were ground in liquid nitrogen using a mortar and pestle, and powdered plant tissue was added directly to 1 mL wash buffer (50 mmol/L Tris/25 mmol/L EDTA/0.36 mol/L sorbitol/0.1 mol/L sodium sulfite/0.1% 2-mercaptoethanol) containing 4 μL RNAse. We raised this solution to a final concentration of 1% (wt/vol) *N*-lauroylsarcosine and 2.5% cetyltrimethylammonium bromide/0.7 mol/L NaCl before proceeding with 24:1 chloroform/isoamyl alcohol extraction. Total genomic DNA was stored in a solution of 10 mmol/L Tris–HCl and 0.5 mmol/L EDTA and quantified using a NanoDrop 1000 (Thermo Fisher Scientific, Waltham, MA).

Amplified Fragment Length Polymorphism (AFLP) marker analysis was performed using a modified version of Vos et al. (1995) described in Sinclair et al. (2008). Adjustments to the protocol for this study include the substitution of 2.6 U *Eco*RI for *Pst*1 as well as *Eco*RI/*Mse*I adaptors in the restriction-ligation mix, and the direct substitution of *Eco*RI primers for *Pst*1 primers in subsequent PCR reactions. The restriction-ligation mix and PCR products were diluted 1:20 prior to pre-selective and selective amplification using an ABI Veriti 96-well thermal cycler (Applied Biosystems, Carlsbad, CA). Selective PCR reactions were pooled and fragments detected on a Beckman CEQ 8000 DNA sequencer using the CEQ Size Standard 400 (Beckman Coulter, Indianapolis, IN). Two primer combinations, *Eco*RI-AGG/*Mse*I-CTG and *Eco*RI-ACC/*Mse*I-CTG, resulted in 134 markers scored between 60 and 400 bp using CEQ 8000 Genetic Analysis System software. DNA markers were double checked manually and replicate samples were run to test the reproducibility of AFLP fingerprints. Individual markers were selected to minimize scoring errors, and final error rates were calculated at ≤3%. Fragment analyses were performed on a total of 135 individuals and an average of 34.5 individuals per site (Table 1). Data files were prepared for genetic analysis using R functions available in AFLPdat (Ehrich 2006).

Summary statistics for AFLP data were calculated using AFLP-SURV 1.0 (Vekemans 2002) and included measures of the proportion of polymorphic loci (PLP) and Nei's gene diversity or expected heterozygosity (H_*j*_). Population structure (*F*_ST_) was also calculated in AFLP-SURV using the method of Lynch and Milligan (1994), assuming Hardy–Weinberg equilibrium and complete outcrossing, and resampled with 10,000 bootstrapped replicates to test significance. We subsequently conducted a hierarchical analysis of molecular variance (AMOVA) implemented in GenAlEx 6.41 with 9999 permutations to partition genetic variation for populations nested in regions (Peakall and Smouse 2006).

The divergence of individuals and population clusters was investigated via non-metric multidimensional scaling ordination methods (MDS) in PRIMER-E 6.1.13 software (Clarke and Gorley 2006). An ordination plot was generated from the genetic distance matrix calculated for all pairwise combinations of individuals in GenAlEx (Wilson et al. 2002; Llorens et al. 2004), and stress was minimized to ≤0.2 for an accurate estimate of plot configuration (Quinn and Keough 2002). ANOSIM, or “analysis of similarities,” was used to test for significant differences among the four *S. hispidum* collection sites. ANOSIM is a non-parametric, multivariate test that calculates R statistics using permutation methods (Clarke 1993). We ran 1000 permutations and compared pairwise *R* values with a global *R*-test statistic to detect significant differentiation within and between northern and southern locations.

### Greenhouse crosses

Whole *S. hispidum* individuals were removed at 5–10 m intervals along walking transects from each of the four sites in October 2007 and transported to the University of Western Australia (UWA). Collections were subsequently transplanted into forestry pots (90 mm × 90 mm × 180 mm) using native soils that remained around plant roots and a standard greenhouse mix (2.5:1:1.5 ratio of composted pine bark, coco peat, and brown river sand; pH 6.5–7), and placed in the UWA greenhouses. Each population was represented by a minimum of 20 adult plants. Mortality rates were low and individuals were replaced when necessary prior to experimental crosses.

Greenhouse collections flowered in spring 2008 over a period of 4 months. Plants from northern sites flowered earlier by 2 or more weeks, but continued to flower as plants from southern populations began to bloom. During that time, triggers of male-phase flowers were removed to hand-pollinate female-phase flowers both within and between populations. Cross-pollinations were conducted following the methods of Burbidge and James (1991) in a closed greenhouse room with no access to potential pollinators, and flowers were not bagged. If crosses were successful, the flowers wilted within a few hours. To the extent possible, hybrids represented reciprocal crosses among multiple pairs of individuals derived from all four sites. Some individuals produced very few flowers or flowered at different intervals, limiting their inclusion in the study. Capsules were removed when dry and typically produced 5–15 seeds each. Once the greenhouse study was completed, a total of 303 successful cross-pollinations were available for viability studies (Table 2). In addition, 25 self-pollinations were conducted for plants from the four populations to test self-incompatibility in this species.

**Table 2:** Number and category of successful cross pollinations of *Stylidium hispidum* representing the four populations. Cross categories include within-population crosses (WP), short-distance hybrids (SD), and long-distance hybrids (LD). Reciprocal crosses are listed separately

♀ × ♂	Category	No. crosses
AV1 × AV1	WP	26
AV1 × AV2	SD	20
AV1 × CPC	LD	15
AV1 × TOR	LD	17
AV2 × AV2	WP	41
AV2 × AV1	SD	19
AV2 × CPC	LD	20
AV2 × TOR	LD	18
CPC × CPC	WP	21
CPC × AV1	LD	14
CPC × AV2	LD	15
CPC × TOR	SD	18
TOR × TOR	WP	18
TOR × AV1	LD	10
TOR × AV2	LD	18
TOR × CPC	SD	13

### Line cross studies

Early fitness was investigated for 764 seeds representing three cross categories: within-population (or intrasite) crosses, short-distance hybrids (3–10 km), and long-distance hybrids (111–124 km). Specifically, 100 seeds were selected from within-population crosses for each northern (AV1 and AV2) and southern (CPC and TOR) population for a total of 400 seeds. A total of 100 seeds corresponded to short-distance hybrids between the two northern Avon populations or the two southern Dwellingup populations. Lastly, 200 seeds were selected to represent long-distance hybrids divided among the four cross combinations between northern and southern populations. Once the original 700 seeds were planted, we increased the sample size based on the availability of selected crosses and greenhouse space. This resulted in an additional 19 seeds for within-population crosses, 29 seeds for short-distance crosses, and 16 seeds for long-distance crosses. Each cross combination (e.g., AV1 × AV2 or AV2 × AV1) in the germination study represented a minimum of 10 maternal plants, and seeds were divided into approximately equal numbers of reciprocal hybrids where each site alternated as the pollen source.

Seeds were soaked overnight in a solution of 100 ppm gibberellic acid (GA_3_) and planted into 50 mm forestry tubes containing the standard greenhouse mix in February 2009. Pots were watered daily in the UWA greenhouses, and germination was monitored weekly over a 4-month period until newly germinated seedlings were no longer visible. The number of surviving seedlings was recorded approximately 6 months later. Prior to planting, average seed weight was recorded for each diallel cross to detect possible maternal effects as well as effects of reciprocal parental combinations. Air-dried seeds were weighed in lots to the nearest 0.01 mg and average seed weight was calculated per cross by dividing the number of seeds in each lot by their total weight. This resulted in a mean of 442 seeds weighed and averaged for each parental combination. Analysis of variance (ANOVA) was used to test the effects of maternal population (AV1, AV2, CPC, and TOR) and experimental cross type on seed mass using JMP version 9.0 (SAS Institute, Cary, NC). The effects of reciprocal pollinations were also investigated. Seed mass was log transformed to meet the assumption of normality.

Binary data for germination and survival for each cross type were analyzed with a binomial distribution and logit link function using PROC LOGISTIC in SAS 9.2 (SAS Institute). Given the potential for maternal effects to influence the survival of progeny, mean seed mass for each reciprocal cross was included as an explanatory variable. Observations in the study had a larger variance than expected given a binomial distribution, and we used the William's method to correct for overdispersion (Allison 2001). The likelihood of germination or survival of different cross categories was estimated by the odds ratio, which describes the probability an event (e.g., germination or survival) occurs divided by the probability that it does not occur. Odds ratios were calculated from the exponent of the parameters of the logistic function to compare pairwise likelihoods of germination or survival for the three categories of within-population, short-distance, and long-distance crosses. For example, we calculated the odds of germination for short-distance crosses relative to long-distance crosses.

## Results

### Genetic diversity and population structure

A total of 135 *S. hispidum* individuals were genotyped for analysis. All but two of the 134 AFLP markers were polymorphic, and values for percent polymorphism and gene diversity were relatively high (Table 1). Estimates of population genetic structure among collection sites revealed significant differentiation (*F*_ST_ = 0.17; *P* < 0.0001). Nested AMOVA partitioned 74% of the total genetic variation within sites (or populations), 5% between sites within regions, and 21% between regions (therefore *Φ*_PT_ = 0.26, *P* = 0.0001).

**Figure 2:**
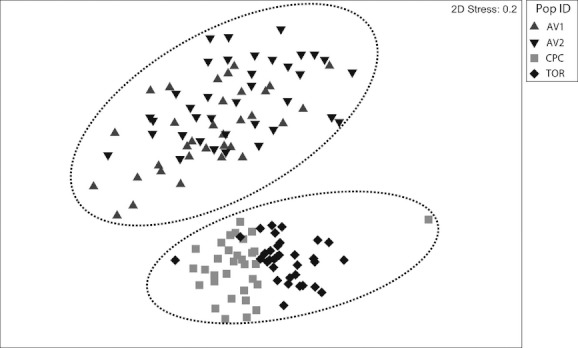
Results of MDS ordination of genetic distance for individual samples of the four populations represented in germination and survival studies. Stress is an estimate of goodness of fit. anosim tests identified two significant clusters (indicated by the dotted lines) that were divided between northern and southern sites (*P* = 0.001).

Non-metric multidimensional scaling separated populations from northern and southern regions, with large overlap among populations within regions (Fig. 2). One outlier was detected among individuals collected at CPC, and may correspond to a rare, long-distance seed dispersal event from an unknown location. The northern and southern regions were significantly differentiated from one another (*R*_ANOSIM_ = 0.623, *P* = 0.001), but ANOSIM did not detect evidence for divergence between populations within either region.

### Experimental crosses

All but two of the experimental pollinations to test self-incompatibility in *S. hispidum* failed, and the crosses that developed seeds set a single seed per fruit. Although greenhouse flowers that were not experimentally pollinated failed to set seeds in almost all cases, it is possible that the rare cases where flowers did set seeds were due to inadvertently transferred pollen. Overall, results support the hypothesis that *S. hispidum* is self-incompatible, but further study may be required to determine the breeding system of this species.

**Figure 3:**
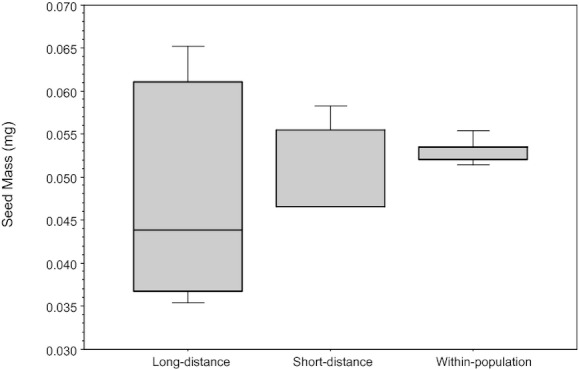
Mean seed mass of the three cross categories for analysis. The boxplot rectangles delimit the 25–75% quartiles.

Results of ANOVA for seed mass detected a significant effect of maternal population regardless of cross type (*F*_3,760_ = 43.25, *P* < 0.0001). The average seed mass for experimental crosses with maternal plants derived from southern sites (CPC and TOR) was 19% greater than seed mass resulting from crosses of maternal plants derived from northern sites (AV1 and AV2). Seed mass did not differ significantly for maternal plants within each set of paired sites. ANOVA also detected an effect of cross type on seed mass (*F*_2,761_ = 53.74, *P* < 0.0001). The average mass of seeds resulting from long-distance crosses was significantly lower than seed mass for either short-distance hybrids or within-population crosses (Fig. 3). Lastly, there was a reciprocal cross effect for all pairs of populations and cross combinations. Long-distance crosses in which AV1 or AV2 plants served as maternal parents resulted in seeds, on average, with lower mass than crosses where CPC or TOR plants served as maternal parents (*F*_1,214_ = 664, *P* < 0.0001). Reciprocal cross effects were also apparent for progeny resulting from short-distance pollinations and separate *t*-tests indicated significant effects of maternal parent on seed mass for both northern and southern sites (*P* < 0.0001).

**Figure 4:**
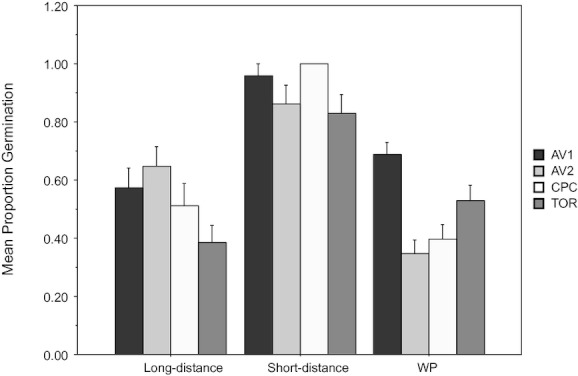
Germination proportions among the three experimental cross distances for each maternal population included in the pollination study. Error bars indicate ±1 standard errors.

**Figure 5:**
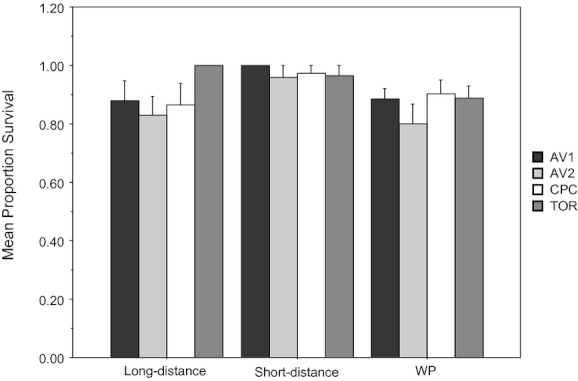
Average survival of seedlings among the three experimental cross distances for each maternal population included in the pollination study. Error bars indicate ±1 standard errors.

At the completion of germination surveys, 57.5% of the 764 seeds had germinated. Of those 439 seedlings, 89% survived during the 10-month study period. Logistic regression analyses of germination data detected a significant effect of cross category resulting in an 11-fold advantage for germination of short-distance crosses compared with within-population crosses and a near 10-fold advantage compared with long-distance crosses (Table 3). Germination success was not significantly different between long-distance and within-population crosses (Fig. 4, Table 3). The effect of the covariate of seed mass on the probability of germination was not significant, although a trend for greater median seed mass was visible for seeds that germinated (Table 4). In survival analysis, we detected an approximate six-fold advantage for progeny resulting from short-distance crosses relative to seedlings of either long-distance or within-population crosses (Table 4, Fig. 5). Once seeds germinated, however, their initial mass had no effect on seedling survival. Similar to germination, the viability of long-distance hybrids did not differ significantly from viability of within-population crosses.

**Table 3:** Odds ratio estimates for germination and survival of within-population (WP) and short- and long-distance crosses (SD and LD, respectively) for *Stylidium hispidum*

Effect	Estimate	95% Wald confidence limits	
Germination			
SD vs. WP	10.986	5.753	20.979
LD vs. WP	1.124	0.809	1.561
LD vs. SD	0.102	0.052	0.200
Survival			
SD vs. WP	5.506	1.629	18.608
LD vs. WP	0.938	0.475	1.855
LD vs. SD	0.170	0.048	0.606

**Table 4:** Results of the logistic regression models for germination and survival of seeds representing the three cross categories of within-population crosses, short-distance and long-distance hybrids. Seed weight represents the mean seed mass of each cross category prior to planting

Source of variation	df	χ^2^ value	*P* value
Germination			
Cross type	2	54.29	<0.0001
Seed weight	1	3.45	0.0631
Survival			
Cross type	2	7.56	0.0229
Seed weight	1	0.31	0.3071

## Discussion

### Population genetic divergence and hybrid performance

Decisions about the sourcing of seeds for ecological restoration are often impeded by a lack of relevant data, especially on the extent and scale of genetic divergence among populations of target species (McKay et al. 2005), as well as an inability to predict the potential for outbreeding depression (Hufford and Mazer 2003). To test if estimates of population genetic divergence reflect hybrid performance, we compared our line cross results with methods proposed for the genetic identification of regions for seed collection via analysis of spatial genetic structure using molecular marker data (Bussell et al. 2006; Krauss and He 2006). Marker analyses of four populations of *S. hispidum* revealed significant variance among populations within regions (5%) and among regions (21%). However, MDS ordination, with support from ANOSIM, detected only two significant clusters split between northern and southern sites. This division corresponded to the greatest cross distance for hybrid comparisons, and predicted two regions or zones for seed transfer among experimental locations. Nested AMOVA results for *S. hispidum* reflect moderate levels of genetic subdivision detected in other endemic, outcrossing plant species from the region (Bussell et al. 2006; Krauss and He 2006; Sinclair et al. 2008, 2010).

We subsequently compared estimates of hybrid performance with estimates of population genetic divergence among the four *S. hispidum* study sites. Significant outbreeding depression was detected for both germination and early survival of intraspecific hybrids between regions at long distances relative to hybrids within regions. This effect was consistent for crosses among 10 or more maternal plants for each of the four populations, with one exception at a southern site (TOR) where early seedling survival of long-distance and short-distance hybrids was equally likely. Thus, our molecular marker data predicted the scale of outbreeding depression among these four sites, providing support for marker use in defining optimally outcrossing seed provenance zones for ecological restoration. Other findings of a significant correlation between genetic diversity and population fitness (Reed and Frankham 2003) indicate that molecular markers can be useful for prediction of adaptive variation in some cases. Further experimentation, however, is required to determine if this result is consistent across the species range.

### Hybrid fitness and outbreeding depression

Outbreeding depression was detected for germination and early survival of seeds resulting from long-distance crosses, despite the known limitations of conducting such studies under relatively benign glasshouse conditions compared with natural conditions. Germination, for example, may depend on environmental cues, such as soil moisture, temperature, and factors, such as the presence of ash after bush fires (Thompson and Ooi 2010). The detection of outbreeding depression in the F1 generation in the greenhouse supports an intrinsic mechanism for reduced fitness at early life stages, rather than an extrinsic, or environmental, mechanism (Edmands 2007). Outbreeding depression in F1 hybrids may reflect the disruption of epistasis and possible chromosome polymorphism. Whereas dysploid evolution is well documented among species of Stylidiaceae (James 1979), some evidence exists for chromosome polymorphism within species – although these studies are rare (Coates et al. 2003). Overall, studies of F1 hybrids represent an important step to estimate hybrid fitness at multiple life stages, and provide data for germination that is difficult to detect in the field.

Predicting the scale of outbreeding depression is critical for the comprehensive application of seed sourcing guidelines in restoration to avoid detrimental consequences of wide outcrossing. Aspects of the biology of *S. hispidum* potentially enable this predictive capacity. Perennial species in the Stylidiaceae often have a post-zygotic mechanism of self-incompatibility (Burbidge and James 1991). Post-zygotic incompatibility is more likely to result in a correlation between population divergence and reproductive compatibility than pre-zygotic incompatibility, which often evolves erratically (Edmands 2002). Frankham et al. (2011) developed a decision tree to predict the probability of outbreeding depression based on species characteristics. They determined that strong environmental variation and population isolation, as well as chromosome differences, increase the probability that outbreeding would lower hybrid fitness. Western Australia is an ancient landscape largely unaffected by major land alterations, such as glaciation, inundation, or volcanic activity for 100 million years (Hopper 2009). Species distributions and spatial genetic structure reflect this low level of disturbance over time, with typically naturally fragmented and disjunct populations systems, and strong isolation-by-distance of gene flow among populations (Hopper and Gioia 2004). This suggests that there is a higher probability of outbreeding depression generally within this diverse flora than in younger and less fragmented landscapes, and this may be generally true of OCBILs (old climatically buffered infertile landscapes) compared with YODFELs (young often disturbed fertile landscapes) (Hopper 2009). Ours is the first study to assess outbreeding depression in this OCBIL landscape, and more studies are required to test this intriguing possibility.

### Inbreeding depression and an optimal outcrossing distance

The line cross study not only detected evidence for outbreeding depression at long distances but also evidence for inbreeding depression within populations. Relative to short-distance crosses, seeds from within-population crosses experienced a significant decline in fitness and their early germination and survival was similar to seeds of long-distance crosses. These results provide evidence for an optimal intermediate outcrossing distance in this species that would balance both inbreeding and outbreeding depression (Waser 1993; Schierup and Christiansen 1996), and that is often seen in plant species. Few studies, however, focus on longer cross distances, and further testing is needed for tiered comparisons of hybrid fitness across a species range (Billingham et al. 2007; Forrest et al. 2011). Our experimental design was limited to within-population, short and long cross distances for the four experimental sites. To better understand the consequences of divergence for hybrid fitness, cross data for short to intermediate distances within the species range are required to determine a maximum distance for seed transfer that would avoid outbreeding depression, as well as a genetic distance that is globally optimal.

Although the overall decline in fitness for inbred and outbred progeny was consistent for all crosses, the percent germination and survival varied among maternal populations. These differences likely reflect different histories, rates of gene flow and dispersal among the four populations, as well as variable rates of inbreeding. For example, long-distance crosses for maternal plants derived from AV2 had higher germination success relative to within-population crosses, and this result may reflect a high fixation index for this population. Seed mass also varied with maternal population and cross category. However, in this case, there was a significant decline in the mass of seeds for long-distance crosses (outbreeding) that was not detected in seed mass of within-population (inbred) crosses. Reciprocal cross effects on seed mass were also apparent, and cytogenetic factors may contribute to outbreeding depression.

### Local provenance versus composite seed mixes

Generally accepted guidelines (e.g., Mortlock 2000; McKay et al. 2005) for the use of local provenance seeds in ecological restoration were developed to avoid negative impacts of maladaptation, inbreeding and outbreeding depression, as well as to maintain areal biodiversity and avoid spatial genetic homogenization or genetic swamping (Krauss and He 2006). Recently, concerns have developed that local provenance is too restrictive, and may result in the introduction of seeds of poor quality to restoration sites as well as the overharvesting of neighboring remnant populations (Broadhurst et al. 2008). Our results for early fitness of *S. hispidum* suggest that there would be a detrimental effect of inbreeding depression if seed collections were limited to few, neighboring plants from single sites. However, inbreeding depression was alleviated via crossing at distances as short as 3 km (range: 3–10 km). This distance falls well within the ∼20 km range for provenance collections currently in practice for post-mining Jarrah forest restoration (Koch 2007). Thus, whereas seed quality remains a key issue for restoration of seed sourcing, the inclusion of genetically diverse collections to avoid inbreeding depression is unlikely to require collection beyond local provenance zones except in rare cases. In cases where neighboring populations are highly fragmented, seed quality may be improved by mixing seeds of multiple fragments from a local provenance. The potential for overharvesting fragments should be assessed by restoration practitioners, and there may be justification to move collections further afield in highly altered landscapes.

Sgrò et al. (2011) support the maintenance of evolutionary potential in restoration, and microevolution should be recognized as a factor for population persistence in changing environments (Rice and Emery 2003). However, there is little evidence to support mixing seed sources over their recommended long distances to achieve sustainable populations. Instead, mixing seeds from highly divergent populations has resulted in other documented cases of outbreeding depression (e.g., Keller et al. 2000; Montalvo and Ellstrand 2001; Forrest et al. 2011). Given the strong outbreeding depression for germination and early survival detected in *S. hispidum*, composite collections at long distances would likely result in a significant decline in hybrid and mean population fitness. At a practical level, data for gene dispersal curves among species are often no more available than data for adaptive differentiation, and applications of this composite seed sourcing method may also limit genetic diversity if collections at long distances are made from few source plants or sites. The selection of a seed collection protocol should consider measures to avoid both inbreeding and outbreeding depression, and be based on the management of risk for individual species and plant communities (Byrne et al. 2011). The strength of the current study is that we have provided insight into the scale at which the local boundary extends from within which a local composite approach to seed collection will optimize breeding effects.

With mounting evidence for outbreeding depression and an optimal outcrossing distance beyond the local population in plant species, we recommend that composite seed mixes for restoration be restricted to a local provenance zone. In doing so, we advise against highly restrictive collection areas, and instead recommend that zones be established with flexible boundaries to represent a mobile collection radius or patch size similar to that in use in Western Australia mine site restoration (Koch 2007). While variation among restoration objectives, life histories of species, and the landscapes within which they occur argues against the development of universal guidelines, practitioners should be generally aware that outbreeding depression is a relevant concern when sourcing seeds for restoration, and must be considered in the context of composite provenances.
